# Supplementary material for: Development of a computer-aided design software for the quantitative evaluation of aesthetic damage
Source: PLoS One. 2019 Dec 18;14(12):e0226322. doi: 10.1371/journal.pone.0226322 (PMC6919621; doi:10.1371/journal.pone.0226322)
Supplement: S1 Code site — 1. (ZIP) [file pone.0226322.s001.zip › [EESC_jr][Projeto]Relatorio_07_21/reports/templates_zip/gentelella-master/vendors/google-code-prettify/README.html]

Javascript code prettifier


Languages : CH

# Javascript code prettifier

## Setup

1. Download a distribution- Include the script tag below in your document

     ```
     >script src="https://google-code-prettify.googlecode.com/svn/loader/run_prettify.js></script>
     ```

     - See Getting Started to configure that URL with options you need.- Look at the skin gallery and pick styles that suit you.

## Usage

Put code snippets in
<pre class="prettyprint">...</pre>
or <code class="prettyprint">...</code>
and it will automatically be pretty printed.

|  |  |  |  |
| --- | --- | --- | --- |
| The original Prettier|  |  | | --- | --- | | ``` class Voila { public:   // Voila   static const string VOILA = "Voila";    // will not interfere with embedded tags. } ```   ``` class Voila { public:   // Voila   static const string VOILA = "Voila";    // will not interfere with embedded tags. } ``` | | | |

## FAQ

### For which languages does it work?

The comments in prettify.js are authoritative but the lexer
should work on a number of languages including C and friends,
Java, Python, Bash, SQL, HTML, XML, CSS, Javascript, Makefiles,
and Rust.
It works passably on Ruby, PHP, VB, and Awk and a decent subset of Perl
and Ruby, but, because of commenting conventions, but doesn't work on
Smalltalk.

Other languages are supported via extensions:

Apollo
Basic
Clojure
CSS
Dart
Erlang
Go
Haskell
Lisp, Scheme
Llvm
Lua
Matlab
MLs:F#, Ocaml,SML
Mumps
Nemerle
Pascal
Protocol buffers
R, S
RD
Scala
SQL
TCL
Latek
Visual Basic
CHDL
Wiki
XQ
YAML

If you'd like to add an extension for your favorite language, please
look at src/lang-lisp.js and file an
issue including your language extension, and a testcase.

### How do I specify the language of my code?

You don't need to specify the language since `prettyprint()`
will guess. You can specify a language by specifying the language extension
along with the `prettyprint` class like so:

```
<pre class="prettyprint lang-html">
  The lang-* class specifies the language file extensions.
  File extensions supported by default include
    "bsh", "c", "cc", "cpp", "cs", "csh", "cyc", "cv", "htm", "html",
    "java", "js", "m", "mxml", "perl", "pl", "pm", "py", "rb", "sh",
    "xhtml", "xml", "xsl".
</pre>
```

You may also use the
HTML 5 convention of embedding a code element inside the
`PRE` and using `language-java` style classes.
E.g. <pre class="prettyprint"><code class="language-java">...</code></pre>

### It doesn't work on <obfuscated code sample>?

Yes. Prettifying obfuscated code is like putting lipstick on a pig
— i.e. outside the scope of this tool.

### Which browsers does it work with?

It's been tested with IE 6, Firefox 1.5 & 2, and Safari 2.0.4.
Look at the test page to see if it
works in your browser.

### What's changed?

See the change log

### Why doesn't Prettyprinting of strings work on WordPress?

Apparently wordpress does "smart quoting" which changes close quotes.
This causes end quotes to not match up with open quotes.

This breaks prettifying as well as copying and pasting of code samples.
See
WordPress's help center for info on how to stop smart quoting of code
snippets.

### How do I put line numbers in my code?

You can use the `linenums` class to turn on line
numbering. If your code doesn't start at line number 1, you can
add a colon and a line number to the end of that class as in
`linenums:52`.

For example

```
<pre class="prettyprint linenums:4"
>// This is line 4.
foo();
bar();
baz();
boo();
far();
faz();
<pre>
```

produces

```
// This is line 4.
foo();
bar();
baz();
boo();
far();
faz();
```

### How do I prevent a portion of markup from being marked as code?

You can use the `nocode` class to identify a span of markup
that is not code.

```
<pre class=prettyprint>
int x = foo();  /* This is a comment  <span class="nocode">This is not code</span>
  Continuation of comment */
int y = bar();
</pre>
```

produces

```
int x = foo();  /* This is a comment  This is not code
  Continuation of comment */
int y = bar();
```

For a more complete example see the issue22
testcase.

### I get an error message "a is not a function" or "opt\_whenDone is not a function"

If you are calling `prettyPrint` via an event handler, wrap it in a function.
Instead of doing
> `addEventListener('load', prettyPrint, false);`

wrap it in a closure like
> `addEventListener('load', function (event) { prettyPrint() }, false);`

so that the browser does not pass an event object to `prettyPrint` which
will confuse it.

### How can I customize the colors and styles of my code?

Prettify adds `<span>` with `class`es describing
the kind of code. You can create CSS styles to matches these
classes.
See the
theme gallery for examples.

### I can't add classes to my code (because it comes from Markdown, etc.)

Instead of `<pre class="prettyprint ...">` you can use a
comment or processing instructions that survives processing instructions :
`<?prettify ...?>` works as explained in
Getting Started

  
  
  

Last modified: Mon Mar 4 14:16:04 EST 2013
